# A Rare Presentation: Cauda Equina Compression Secondary to an L1 Burst Fracture in Osteoporosis

**DOI:** 10.7759/cureus.21425

**Published:** 2022-01-19

**Authors:** Leong Yen Hsin, Huang Yilun

**Affiliations:** 1 Department of Orthopaedic Surgery, Sengkang General Hospital, Singapore, SGP

**Keywords:** rare presentation, variant anatomy, osteoporosis, l1 burst fracture, cauda equina compression

## Abstract

Cauda equina syndrome (CES) rarely occurs in upper lumbar spinal pathologies above L2. Osteoporosis is a consideration in determining the operative approach. We report a case of CES as a result of an L1 burst fracture in an osteoporotic lady with schizophrenia.

A 74-year-old schizophrenic lady presented with traumatic lower back pain with no neurological deficit. Due to her psychiatric condition, the clinical assessment was challenging. On day 3 of admission, there was an acute total loss of motor function over bilateral L2-L3 myotomes to MRC grade 0/5, progressively involving bilateral L2-S1 myotomes symmetrically. There was associated symmetrical bilateral lower limb hypotonia, areflexia, acute urinary retention, and absence of anal tone and bulbocavernosus reflex. Magnetic resonance imaging (MRI) reported a severe L1 compression fracture with retropulsion and cauda equina compression. Conus medullaris terminated at T12. An L1 anterior corpectomy and decompression with T11-L3 posterior instrumentation and stabilization were performed. Intraoperatively noted osteoporotic bone. Postoperatively, motor function improved to MRC grade 4/5 over bilateral L4-S1 myotomes by postoperative day 15 with rehabilitation.

A variant in anatomy may result in a high differentiation of the conus medullaris into the cauda equina. Thus, an L1 burst fracture may, on rare occasions, result in CES instead of conus medullaris syndrome. Special attention needs to be given to psychiatric patients who are unable to provide a good history and comply with a physical examination. MRI remains the diagnostic gold standard for CES. Early diagnosis and early surgical decompression are recommended for maximum functional recovery. Osteoporosis further complicates the operative intervention as both the anterior and posterior approaches must be adapted for better stabilization and surgical outcome. Early initiation of rehabilitation is crucial for postoperative functional recovery.

## Introduction

Cauda equina syndrome (CES) is uncommon and rarely occurs in upper lumbar spinal pathologies above the L2 level [1]. CES represents only 5% of all cases of spine trauma [2]. Osteoporosis is a consideration in determining the operative approach. We report a case of CES as a result of an L1 burst fracture in an osteoporotic lady with schizophrenia.

This article was previously presented as a poster at the 8th International Malaysia Spine Society Scientific Congress held on the 7th and 8th of August 2021.

## Case presentation

A 74-year-old schizophrenic lady presented with lower back pain following a fall. She had no neurological deficits at the time of presentation. On day 3 of admission, she developed an acute total loss of motor function over bilateral L2 and L3 myotomes to MRC grade 0/5, which progressed to involving bilateral L2-S1 myotomes symmetrically within 12 hours. There was associated symmetrical bilateral lower limb hypotonia, areflexia, and acute urinary retention. Anal tone and the bulbocavernosus reflex were also absent.

**Figure 1:**
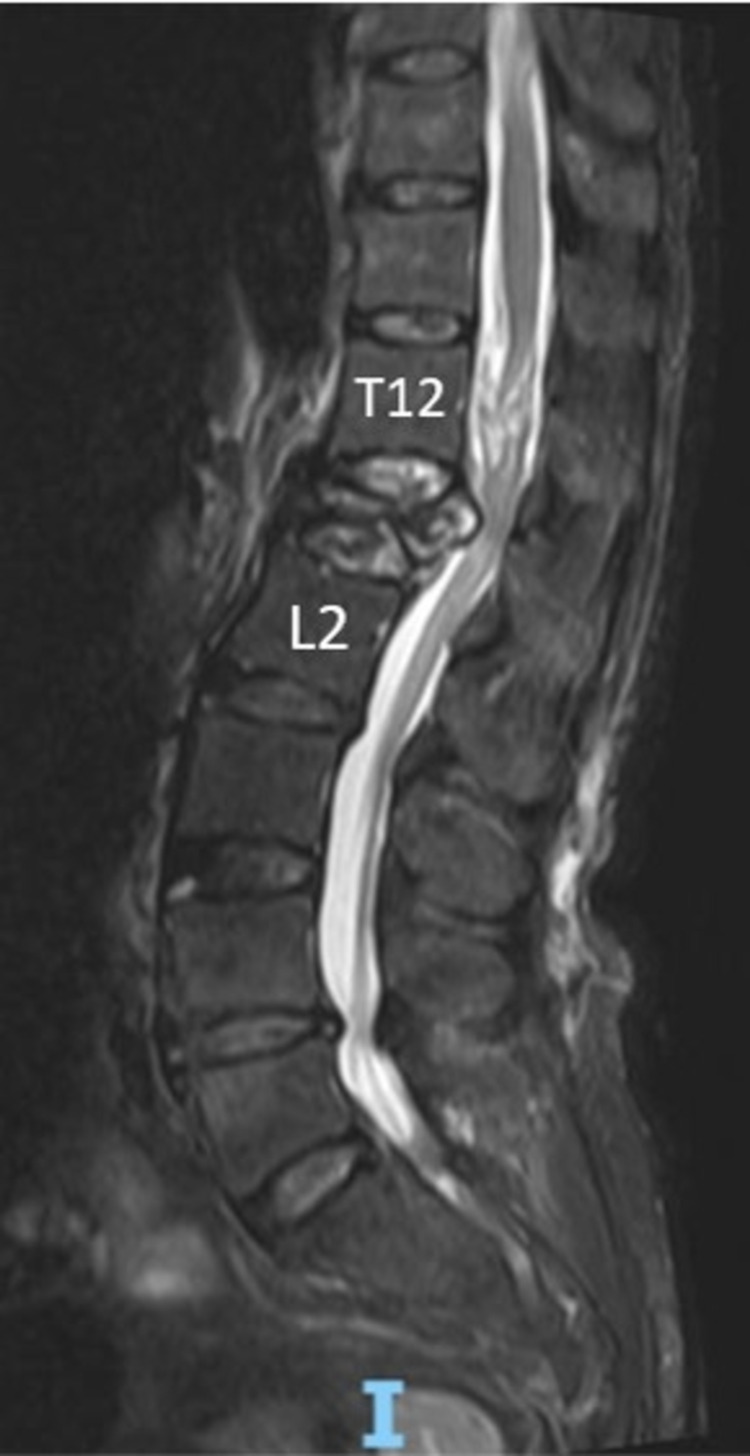
Preoperative T2-weighted MRI of the lumbar spine - sagittal view

**Figure 2:**
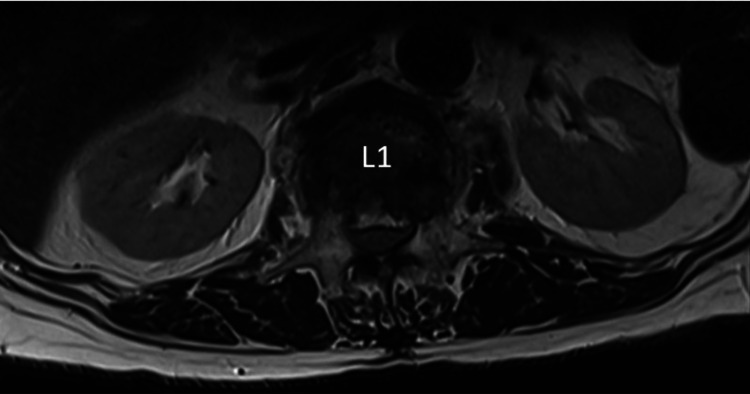
Preoperative T2-weighted MRI of the lumbar spine - axial view

A plain radiograph noted a severe L1 compression fracture. MRI reported a severe L1 compression fracture with retropulsion and compression of the cauda equina. Conus medullaris terminated at the T12 level and there were subacute ascending myelopathic changes (Figures 1, 2).

**Figure 3:**
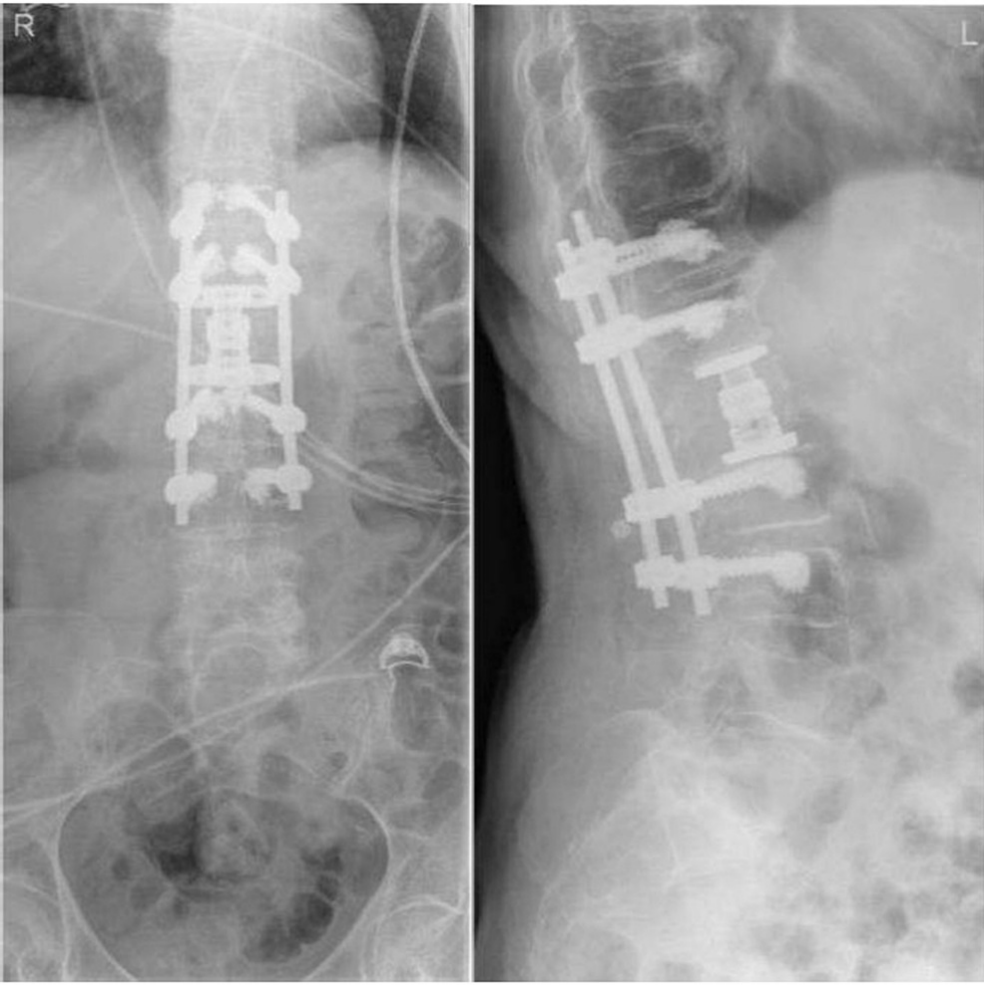
Postoperative radiographs of the lumbar spine

An L1 anterior corpectomy and decompression with T11-L3 posterior instrumentation and stabilization were performed. Intraoperatively, she was noted to have osteoporotic bone and an L1 burst fracture with a retropulsed bone fragment causing cauda equina compression. Postoperative radiographs were satisfactory (Figure 3). Histology reported no evidence of malignancy.

Postoperatively, she had a good neurological recovery with rehabilitation. Motor function improved to MRC grade 4/5 over bilateral L4-S1 myotomes by postoperative day 15.

## Discussion

Traumatic spinal injury at the level of L1 rarely results in CES as the conus medullaris terminates at the level of L1 and L2 [1,3,4]. The clinical symptoms of CES typically includes lower back pain, sciatica, saddle anesthesia, bilateral lower limb sensory and motor disturbances, and bowel bladder dysfunction [1,5]. Our patient initially presented to us with traumatic lower back pain without neurological deficit, and was only noted to have an acute total loss of motor function over bilateral L2-S1 myotomes on day 3 of admission. Due to her psychiatric condition, history-taking and physical examination were challenging. Reaching a diagnosis based on the unclear history and effort-dependent clinical findings was a hurdle. The presence of lower motor neuron signs (hypotonia, areflexia, acute urinary retention, absence of anal tone, and bulbocavernosus reflexes) raised the suspicion of a true spinal pathology. MRI confirmed the diagnosis of CES due to severe L1 compression fracture with retropulsion and incidentally noted an anatomical variant in which the conus medullaris terminated at the level of T12. To date, there is no other case reported on CES occurring at the level of L1.

The operative approach was further complicated by the osteoporotic bone noted intraoperatively. In general, posterior decompression is often adequate, unless there is a lesion in the anterior spine as well, such as vertebral destruction, neoplasm, or large abscess [6]. In this case, T11-L3 posterior instrumentation and stabilization were performed. Intraoperatively, there was an L1 burst fracture with retropulsed bone fragments, causing cauda equina compression. The bone was severely osteoporotic. Thus, the decision was made to proceed with an anterior approach for better stabilization. A left lateral thoracotomy incision in layers was made, followed by the excision and extraction of the ribs, and subsequently the excision of the diaphragm and extrapleural approach to the spinal level. L1 anterior corpectomy and decompression were done in a piecemeal style with preservation of the posterior dural and neural elements. An expandable cage filled with autograft bone chips was inserted. Postoperative radiographs were satisfactory.

Literature is equivocal on the timing of decompression. In a retrospective case series, it was found that there was complete resolution of bowel and bladder symptoms when the decompression was performed within 48 hours of the onset of symptoms [7]. However, another study found no correlation between the preoperative duration of symptoms and functional recovery [8]. Early initiation of rehabilitation was found to have a significant association with walking ability at discharge [9]. In this case, decompression and stabilization were performed within 12 hours of the onset of symptoms. The patient showed good neurological recovery with the improvement of motor function to MRC grade 4/5 over bilateral L4-S1 myotomes by postoperative day 15 with rehabilitation. Though there is no consensus on the urgency of surgery, early diagnosis and early surgical decompression are recommended for maximum functional recovery [10,11].

## Conclusions

A high differentiation of the conus medullaris to the cauda equina may result from variant anatomy. Thus, an L1 burst fracture may, on rare occasions, result in CES instead of conus medullaris syndrome. Special attention needs to be given to psychiatric patients who are unable to provide a good history and comply with a physical examination. MRI remains the diagnostic gold standard for CES. Early diagnosis and early surgical decompression are recommended for maximum functional recovery. Osteoporosis further complicates the operative intervention as both the anterior and posterior approaches must be adapted for better stabilization and surgical outcome. Early initiation of rehabilitation is crucial for postoperative functional recovery.
